# The Princess

**Published:** 1889-04-27

**Authors:** 


### The Princess.

It was a happy day for this country when the Prince of Wales, like Isaac the son of Abraham, travelled far in search of a wife. It is always said, and doubtless with truth, that the Danish princess who is now an English princess was brought up with extreme simplicity. How thankful she must often have been since she came to the land of her adoption for that early discipline! The German princes all learn some sort of handicraft. Prussian history affords many illustrations of the mutability of human affairs; and it is always possible that any individual German prince may, some day or other, have reason to be glad that he knows how to use the workman's tools. Those Northern countries practise a stern simplicity of outlook and manners which is a standing rebuke to much of the weak and vulgar effeminacy so common in this country; common not merely among the noble and the wealthy, but also among many of the middle classes, whose experience should have inspired them with better sense.

The Princess has proved herself a lady of rare excellence, and, so far as can be seen, admirably adapted to be the Prince's wife and the Queen's daughter-in-law. That she has won the hearts of the British people, all men will unitedly affirm. To be the successful wife even of a bishop is no easy matter, as Mrs. Proudie's conspicuous failure abundantly proved. But to have won the heart of the Prince, the affection of the Queen, and the enthusiastic devotion of the whole people of Great Britain is at least a sufficient achievement for one Royal lady's lifetime. Perhaps the simple virtues of the Danish home have proved their wearing qualities in the Royal household that is the model for all the households of this vast empire. The story of the Princess of Wales is not merely that of the Prince's wife or of the mother of his children: she has an individuality all her own. If it be of the modest, quiet, and home-loving kind, is it any the less dignified and sweet on that account? Is it not all the more so? Is it difficult to imagine any lady more suited to the peculiar times in which the Prince's lot has been cast than the Princess. Gentle, tender-hearted, loving the people, living among them, understanding their sorrows, sharing their pleasures, making herself—to use a homely phrase—so entirely "at home," she has endeared herself and her very name to every home in the land.

Not less to the sick than to the well is the Princess a familiar figure, and her smile a coveted grace. During the quarter of a century in which she has been in this country every kind of charitable institution has borne witness to her sympathetic zeal. In an especial manner has she devoted herself to hospitals for women and children, and to all kinds of institutions which have for their object the lightening of the burdens of the weaker sex, and the helping of those to whom life has proved too fierce a conflict. She has joined hands with those brave and noble women who, on the battle-field, in the workhouse, at the hospital, in the fever den, have given themselves up "willing sacrifices" to the highest ideals of duty and of service. What other way of life could be nobler, more truly womanly than this? The triumphs of the Assembly and of the Court may last a brief hour and are then forgotten: the good deeds that spring from a good heart are remembered to the remotest generation.

The impulse given by the Prince and Princess to all kinds of philanthropic work has not been of that indiscriminating character which, whilst it does a certain amount of good, may more than counterbalance the good by a foolish and injurious selection of objects to be encouraged. Counsel has constantly been taken of those who best knew how to advise on practical affairs. How seldom, it has happened that either of their Royal Highnesses has had to withdraw name or patronage when once they had been given! One highly-important practical consequence of this has been the greater furtherance of every meritorious institution, and the checking and enfeebling of all kinds of worthless causes which have deserved only extinction. The survey of charitable work and its progress in this country during the last twenty-five years affords a truly remarkable spectacle. At the beginning of that period, although much had been done, yet much still remained of confusion, chaos, cruelty, selfishness, incapacity, and utter worthlessness, even among charitable institutions themselves. The state of many of the workhouses and their hospitals—to take a single example—was a scandal against decency and a disgrace to civilisation. All that has been changed as completely as if it had never been known. The workhouse infirmaries of to-day are such that no man, nor even king, need desire a better hospital in time of sickness or of danger to life or limb. Neither the Queen, the Prince, nor the Princess will think of taking the entire credit of this improved condition of affairs to themselves. But it is quite certain that both Her Majesty and her Royal children have contributed by every possible means in their power to so great and beneficent a change. The Victorian era will be known in history for many vast and splendid achievements; but for none will it be more gratefully remembered than for its remarkable ameliorations of the condition of the poor and the sick. Those ameliorations have always had the fullest sympathy and, wherever possible, the practical help of the Prince and Princess of Wales.

